# Working hours and self-rated health over 7 years: gender differences in a Korean longitudinal study

**DOI:** 10.1186/s12889-015-2641-1

**Published:** 2015-12-23

**Authors:** Seong-Sik Cho, Myung Ki, Keun-Hoe Kim, Young-Su Ju, Domyung Paek, Wonyun Lee

**Affiliations:** Department of Occupational and Environmental Health, Graduate School of Public Health, Seoul National University, 1 Gwanak-ro, Gwanak-gu Seoul, South Korea; Department of Occupational and Environmental Medicine, Konkuk University Chungju Hospital, 82 Gukweondae-ro Chungju, Chungbuk, South Korea; Department of Preventive Medicine, School of Medicine, Eulji University, 143-5, Yongdu-dong, Jung-gu, Daejeon, South Korea; Department of Occupational and Environmental Medicine, Cheongju Medical Center, 48 Heungdeok-ro, Seowon-gu, Cheongju city, Chungbuk South Korea; Department of Occupational and Environmental Medicine, Hallym University Sacred Heart Hospital, 22 Gwanpyeong-ro 170 beon-gil, Dongan-gu, Anyang, Kyeonggi South Korea

**Keywords:** Working hours, Self-rated health, Gender

## Abstract

**Background:**

To investigate the association between long working hours and self-rated health (SRH), examining the roles of potential confounding and mediating factors, such as job characteristics.

**Methods:**

Data were pooled from seven waves (2005–2011) of the Korean Labour and Income Panel Study. A total of 1578 workers who consecutively participated in all seven study years were available for analysis. A generalized estimating equation for repeated measures with binary outcome was used to examine the association between working hours (five categories; 20–35, 36–40, 41–52, 53–68 and ≥69 h) and SRH (two categories; poor and good health), considering possible confounders and serial correlation.

**Results:**

Associations between working hours and SRH were observed among women, but only for the category of the shortest working hours among men. The associations with the category of shortest working hours among men and women disappeared after adjustment for socioeconomic factors. Among women, though not men, working longer than standard hours (36–40 h) showed a linear association with poor health; OR = 1.41 (95 % CI = 1.08–1.84) for 52–68 working hours and OR = 2.11 (95 % CI = 1.42–3.12) for ≥69 working hours. This association persisted after serial adjustments. However, it was substantially attenuated with the addition of socioeconomic factors (e.g., OR = 1.66 (95 % CI = 1.07–2.57)) but only slightly attenuated with further adjustment for behavioural factors (e.g., OR = 1.63 (95 % CI = 1.05–2.53)). The associations with job satisfaction were significant for men and women.

**Conclusions:**

The worsening of SRH with increasing working hours only among women suggests that female workers are more vulnerable to long working hours because of family responsibilities in addition to their workload.

**Electronic supplementary material:**

The online version of this article (doi:10.1186/s12889-015-2641-1) contains supplementary material, which is available to authorized users.

## Background

South Korea (hereafter Korea) had the longest working hours between 1980 and 2007 and the second longest since 2007 after Mexico among the Organization for Economic Cooperation and Development countries [1]. In principle, legal working hours in Korea are 40 h per week based on a five-day working week system with the establishment of a legal limitation of working hour per week – not to exceed 52 h. This legislation was introduced in 2004 from public sector and companies with over 1000 full-time workers and gradually extended to companies with five full-time workers or more in 2011. Despite the five-day working policy, working long hours has still been routine culture in Korea, partly because of rising job insecurity and high work pressure.

Globally, and recently in Korea, previous studies reported, with sufficient evidence, the negative effects of long working hours on health, including depressive symptoms [2–4], sleep disturbance [5, 6] and cardiovascular disease [7–10], which is often best recognized by the word *karoshi* (death from overwork). Inconsistency in associations of long working hours has been observed for diabetes [10], health behaviour [11], obesity [12], suicide [13], and self-rated health (SRH) [14]. Much of this inconsistency may be attributable to the complexity involved in defining working hours in the modern working environment. With the expansion of precarious work over recent decades, the components of long working hours have become increasingly less standardized and have become a function of many factors: job insecurity, autonomy, enjoyment and self-fulfilment, high demands, work schedule, and job satisfaction [8, 15–17].

Despite the advances in elucidating the details of the nature of working hours, unanswered questions remain on the distinction between the combined and separate effects of working hours and other job characteristics. Earlier studies reported that the effects of working hours were in operation in combination with, for example, length of rest break [18] and work flexibility [17] rather than alone. Similarly, long working hours have been proposed as a predisposing factor for shift work tolerance [19]. Moreover, some recent studies have shown that some workers are forced to take part-time jobs because of the reasons for constrained competence such as health problems [3, 20]. In line with this, when shorter working hours were concurrent with rising work intensity, it had no noticeable positive effects [21]. Such findings suggest that the benefits of short working hours may not be fully measurable in an unfavourable situation where the working environment becomes increasingly insecure and competitive like in Korea. Though variability in working hours has increased across both sides of standard working hours (40 h in Korea), to date, research on short working hours has been largely neglected. Further, there is uncertainty as to how long working hours negatively affects health. Long working hours tend to co-occur with unhealthy behaviours and could be a proxy for lower socioeconomic status [11, 22]. Though, to some extent, prior studies included either behavioural or socioeconomic factors, it is still unclear whether long working hours have independent effects and, if not, what factors mainly account for the effects of long working hours. Lastly, it is well-known that poor health plays an active role in constructing the gender gap in paid employment and employment transition and generally plays a more significant role for women than for men [23]. This implies that women may have higher risks from long working hours, but the evidence is scarce.

Given insufficient understanding of some details, we investigated the association between working hours and SRH using a large Korean longitudinal data. This study aimed to examine whether (1) working hours (both short and long working hours) are associated with SRH, with particular attention to gender differences, (2) the associations are influenced by other domains of job characteristics such as job satisfaction and work shift, and (3) the associations are independent of socio-demographic and behavioural factors.

## Methods

### Study population

The Korean Labour and Income Panel Study (KLIPS) is an ongoing longitudinal survey of a nationally representative sample. The same individuals were re-interviewed each year about various social and economic activities, including employment, income, education, and training. The study subjects used here were drawn from seven waves of the KLIPS between 2005 and 2011 (from the 8th to the 14th wave), where relevant information was available. Initially, 4086 workers who had no missing values on SRH and working hours were included. After exclusion of individuals who did not participate in all of the seven consecutive years, a total of 1578 workers remained for analysis of balanced panel data to make equal contributions from all of the participants. Ethical approval was obtained from the Institutional Research Board at Konkuk University Hospital, and secondary data analysis was performed without identifying personal information.

### Measures

As a health measure, SRH over the last 12 months was used. A 5-point scale of SRH was collapsed into two categories: a rating of “very good” or “good” health status was identified as “good health” and a rating of “fair”, “poor”, or “very poor” health status was regarded as “poor health”. For participants who worked on a regular basis, working hours were calculated by asking a set of two questions: “What are your current regular working hours for a week excluding meal times?” and “On average, how many hours do you work overtime per week?” For workers who did not work on a regular basis, working hours were assessed by asking: “How many hours do you work in a week on average?” Working hours with a minimum of at least 20 h were categorized into five groups to reflect the current status of working hours authorized by Korean law, and the significance for each category is indicated in parenthesis; 20–35 h (less than standard working hours), 36–40 h (standard working hours), 41–52 h (overtime within legally permitted working hours per week excluding weekend work), 53–68 h (overtime within legally permitted working hours per week including weekend work) and 69 h or more (legally prohibited working hours in any circumstances). Binary variables were generated for working days per week (i.e. ≤5 days or >5 days), shift work and job satisfaction (i.e. yes or no). Based on the Korean Standard Industrial Classification, industrial sectors were grouped into five categories as follow; agriculture, fishing, mining and construction; manufacturing; retail, restaurant and hotel; financing, estate, transportation and information; health, social, educational and household services. Initially, occupations were classified into 10 major groups according to the Korean Standard Classification of Occupation and then collapsed into three categories; manager and professional (managers, professionals, technicians and associate professionals), white collar workers (clerks, service and sales workers) and manual workers (skilled agricultural and fishery workers, craftsmen and related workers, plant and machine operators and elementary occupations).

Relevant socio-demographic and behavioural covariates were identified from previous studies. Three categories of educational attainment were distinguished: middle school or less, high school, undergraduate and postgraduate. Monthly income was based on a single question about the amount of wages earned in the previous month; data were then grouped into quintiles. Employment status was grouped into three categories: regular, temporary and day labour, on the grounds of the contract period. Smoking habits were classified into two categories: current smoker vs ex- and non-smoker. Similarly, alcohol consumption was graded into two categories: current alcohol drinkers vs ex- and non-drinkers.

### Statistical analysis

To explore the basic characteristics of the study population and basic associations, simple descriptive statistics were reported from the data pooled over seven years. For the analyses of association between working hours and SRH, the generalized estimating equation was applied to adjust for confounders and to consider the serial correlation between repeated measures within an individual. To clarify the temporal intervals, SRH in *t* + 1 year was secured to be ahead of predictors in the preceding *t* year (a lag time of 1 year). Application of a set of covariates for adjustments was identified using the Directed Acyclic Graph (DAG) approach [24], which is able to achieve a model selection accounting for a conceptual causal relationship. When a list of variables becomes sufficient enough to minimize confounding and selection bias under a causal assumption [25], DAG presents a model described as ‘minimal sufficient adjustment set’ of covariates. Models were fitted with different adjustments for covariates and stratified by gender. Model 1 was fitted only for working hours. Model 2 identifying a minimally sufficient adjustment set included age, working days, monthly wage, educational level, shift work, job status, along with long working hours. Then, additional to the covariates defined in the Model 2, further adjustment was made for behavioural factors (smoking and alcohol) and job satisfaction (Model 3). We tested this model with DAG and found that it is sufficient to build a causal structure between long working hours and SRH (Additional file 1). Statistical inference applied to panel data depends on time period in use, as Individual effects change over time; that is, for example, SRH declines with ages. It is therefore argued that the time-varying nature of SRH can be better modeled by introducing a longer panel [26, 27]. Panels have lasted for one to seven waves in the current study and, to minimize the period-dependent heterogeneity, we included only individuals who provided entire information on the variables specified in the Model 3 over all seven years (six observations per each individual, as the associations of predictors with SRH in the current study was defined to occur over a period of year *t* to *t* + 1). We also conducted analyses involving a different timescale with cross-sectional association (no time lag) and a similar magnitude and pattern of associations was observed (data not presented). To assess potential bias associated with the categorization, we repeated the same analysis with a slightly different definition of SRH; good (“very good”, “good” or “fair”) versus poor (“poor” or “very poor”) health status and associations were similar (data not presented) to the results presented here. Statistical analyses were conducted with STATA ver13 (StataCorp LP, Texas, USA) and an online program “dagitty” [28] for DAG analysis.

## Result

The study population comprised 6432 observations from 1072 men and 3036 observations from 506 women (Table 1). Men and women were similar in age distribution. Women had a higher proportion of poor health than men (38.9 vs 30.7 %). Approximately, 75 % of men and 70 % of women worked more than the standard working hours (40 h per week) but more women worked longer than 5 days per week than men. Compared to men, women were in less secure jobs, with more of them in temporary or day labour jobs, but women were less likely to do shift or night work and were more in the white collar workers category. Women were less educated and 27.2 % of women and 14.0 % of men did not finish high school.

**Table 1:** Socio-demographic characteristics of the study sample pooled over seven years (2005–2011) in the Korean Labour and Income Panel Study

	Men	Women		Total
	N (%^a^)	N (%)	*p*-value^b^	%
Number of individuals	1072(67.9)	506(32.1)		100.0
Number of observations	6432(67.9)	3036(32.1)		100.0
Age				
20–45	4137(64.3)	1957(64.5)		64.4
≥46	2295(35.7)	1079(35.5)	0.89	35.6
Self-rated health^c^				
Good	4458(69.3)	1855(61.1)		66.7
Poor	1974(30.7)	1181(38.9)	<0.001	33.3
Working hours				
20–35 h	206(3.2)	197(6.5)		4.3
36–40 h	1414(22.0)	739(24.3)		22.7
41–52 h	2617(40.7)	1290(42.5)		41.3
53–68 h	1564(24.3)	611(20.1)		23.0
≥69 h	631(9.8)	199(6.6)	<0.001	8.8
Working days				
≤5 days	3482(54.1)	1341(44.2)		51.0
>5 days	2950(45.9)	1695(55.8)	<0.001	49.1
Job status				
Regular	5553(86.3)	2426(80.0)		84.3
Temporary	227(4.3)	314(10.3)		6.2
Day labor	602(9.4)	296(9.8)	<0.001	9.5
Work Schedule				
Day work	957(14.9)	200(6.6)		12.2
Shift/night work	5475(85.1)	2836(93.4)	<0.001	87.8
Job satisfaction				
Yes	5865(91.2)	2839(93.5)		92.0
No	567(8.8)	197(6.5)	0.001	8.1
Industry				
Agriculture, fishing, mining and construction	1068(16.6)	113(3.7)		12.5
Manufacturing	1994(31.0)	697(23.0)		28.5
Retailer, restaurant and hotel	647(10.1)	596(19.7)		13.1
Financing, estate, transportation and information	1050(16.3)	317(10.5)		14.5
Health, social, educational and household services	1667(25.9)	1309(43.2)	<0.001	31.5
Occupation				
Manager and professional	1537(24.1)	792(26.1)		24.7
White collar workers	1650(25.8)	1269(41.8)		40.0
Manual workers	3203(50.1)	975(32.1)	<0.001	44.3
Education				
Less than high school	899(14.0)	825(27.2)		18.2
High school	2414(37.5)	952(31.4)		35.6
University or more	3119(48.5)	1259(41.5)	<0.001	46.2
Monthly wage				
1 (lowest quintile)	270(4.2)	810(26.7)		11.4
2	565(8.8)	876(28.9)		15.2
3	1238(19.3)	523(17.2)		18.6
4	1926(29.9)	425(14.0)		24.8
5 (highest quintile)	2433(37.8)	402(13.2)	<0.001	29.9
Smoking				
Yes	3586(55.8)	35(1.2)		38.2
No	2846(44.3)	3001(98.9)	<0.001	61.8
Drinking				
Yes	5453(84.8)	1624(53.5)		74.8
No	979(15.2)	1412(46.5)	<0.001	25.3

^a^%: row frequency apart from number of observations and individuals

^b^ *p*-value was obtained from Chi-square test

^c^For self-rated health, data were pooled over a period between 2006 and 2011, but for all other measures between 2005 and 2010

Socio-demographic differences across a range of working hours were presented (Table 2). Men were more in the categories of longer working hours than women. In general, when workers were in unfavourable situations (e.g. poor health, less than university level education, and receiving a lower monthly wage), they worked longer working hours. The gap in working hours across categories of these characteristics was larger among women than men. For example, women with less than high school education who worked over 52 working hours were 39.9 % and the proportion of those with university education was down to 12.7 %. However, the corresponding figures among men were 36.5 and 27.5 %, and the gap was far narrower than that of women. However, those with a better labour market position such as regular and day workers were likely to work longer hours, which was particularly apparent among men.

**Table 2:** Sample characteristics across a range of working hours by men and women pooled over seven years (2005–2011) in the Korean Labour and Income Panel Study

	Men					Women				
	20–35 h	36–40 h	41–52 h	52–68 h	≥69 h	20–35 h	36–40 h	41–52 h	52–68 h	≥69 h
Gender	3.2	22.0	40.7	24.3	9.8	6.5	23.3	42.5	20.1	6.6
Age										
20–45	1.9	20.9	41.0	26.6	9.5	4.2	28.1	45.7	17.1	5.0
≥46	5.5	24.0	40.0	20.1	10.3	10.6	17.6	36.7	25.7	9.5
Self-rated health										
Good	2.4	22.0	41.9	24.5	9.3	4.9	25.7	46.0	18.5	5.0
Poor	5.1	21.9	37.9	24.0	11.0	9.1	22.3	36.9	22.7	9.1
Working days										
≤5 days	5.3	38.7	36.5	13.2	6.3	11.5	49.7	29.6	7.6	1.6
>5 days	0.7	2.3	45.6	37.5	13.9	2.5	4.3	52.7	30.0	10.4
Job status										
Regular	1.2	21.3	41.7	25.4	10.4	3.1	25.0	45.4	20.3	6.3
Temporary	8.3	21.7	35.7	19.9	14.4	12.4	22.9	34.1	21.3	9.2
Day labor	19.4	28.2	33.4	16.5	2.5	28.0	20.6	27.7	17.6	6.1
Work schedule										
Day work	1.0	13.3	33.8	27.0	25.0	3.5	36.0	38.5	20.0	12.0
Shift/night work	3.6	23.5	41.9	23.9	7.2	6.7	24.2	42.8	20.1	8.2
Job Satisfaction										
Yes	2.3	22.6	41.6	24.0	9.5	5.8	24.8	43.4	19.7	6.3
No	12.2	15.3	31.8	27.9	12.9	16.2	17.8	29.4	26.9	9.6
Education										
Less than high school	11.5	16.4	35.7	24.7	11.8	13.1	17.0	30.1	29.1	10.8
High school	2.7	17.5	38.0	27.9	14.0	5.5	19.0	41.8	24.7	9.0
University or more	1.2	27.1	44.2	21.5	6.0	2.9	33.2	51.2	10.8	1.9
Monthly wage										
1 (lowest quintile)	21.1	14.4	18.9	15.2	30.4	18.3	21.2	36.3	20.1	4.1
2	8.7	16.1	36.1	24.6	14.5	3.3	17.6	39.4	28.8	11.0
3	3.7	18.2	41.4	26.8	9.9	2.3	24.7	41.5	21.8	9.8
4	1.4	19.3	40.2	29.8	9.4	1.4	35.8	50.8	9.7	2.4
5 (highest quintile)	1.1	28.3	44.2	19.7	6.7	0.5	32.8	54.2	10.2	2.2
Smoking										
Yes	3.8	20.2	39.7	26.4	9.8	5.7	25.7	28.6	22.9	17.1
No	2.4	24.2	42.0	21.6	9.8	6.5	24.3	42.7	20.1	6.4
Drinking										
Yes	3.1	22.1	41.2	24.2	9.4	5.7	24.6	42.7	20.8	6.3
No	0.6	3.2	5.8	3.8	1.8	7.4	24.1	42.2	19.4	6.9

The same number of observations were used as identified in Table 1

The bivariate relationship between predictors and SRH is shown in Table 3. The level of working hours had a U-shaped association with a higher risk of poor health. That is, in general, the highest risk of poor health occurred among those who worked the shortest hours (20–35 h), followed by those who worked the longest hours (≥69 h). The lowest risk was observed in the range of 41–52 working hours both in men and women. Workers on a regular contract were less likely to have poor health, but no significant differences were observed between categories of work schedules. The pattern of association between socioeconomic factors and poor health status showed a gradient from more to less advantaged for both education and wage, which was common in men and women. There was a higher risk of poor health with smoking, which was more pronounced among women, while the trend for drinking was in the opposite direction: i.e. those who drank more had better SRH.

**Table 3:** Bivariate associations of working hours and other measures with self-rated health by men and women pooled over seven years (2005–2011) in the Korean Labour and Income Panel Study

	Men			Women		
	Good health	Poor health	*P*-value^a^	Good health	Poor health	*P*-value
Age						
20–45	73.7	26.3		71.0	29.0	
≥46	61.4	38.7	<0.001	43.2	56.8	<0.001
Working hours						
20–35 h	51.0	49.0		45.7	54.3	
36–40 h	69.4	30.6		64.4	35.6	
41–52 h	71.4	28.6		66.2	33.8	
53–68 h	69.7	30.3		56.1	43.9	
≥69 h	65.6	34.4	<0.001	46.2	53.8	<0.001
Work day						
≤5 days	69.6	30.4		61.9	38.1	
>5 days	69.0	31.1	0.56	60.5	39.5	0.42
Job status						
Regular	71.6	28.4		65.9	34.1	
Temporary	64.3	35.7		48.1	51.9	
Day labor	50.5	49.5	<0.001	35.8	64.2	<0.001
Work Schedule						
Day work	67.4	32.6		51.0	49.0	
Shift/night work	69.6	30.4	0.16	61.8	38.2	0.002
Job satisfaction						
Yes	70.6	29.4				
No	56.3	43.7	<0.001			<0.001
Education						
Less than high school	52.5	47.5		37.2	62.8	
High school	67.9	32.1		62.7	37.3	
University or more	75.3	24.8	<0.001	75.5	24.5	<0.001
Monthly wage						
1 (lowest quintile)	50.0	50.0		44.3	55.7	
2	58.2	41.8		27.9	30.3	
3	66.3	33.7		68.8	31.2	
4	69.5	30.5		77.2	22.8	
5 (highest quintile)	75.4	24.6	<0.001	72.1	27.9	<0.001
Smoking						
Yes	67.9	32.1		37.1	62.9	
No	45.4	41.7	0.006	61.4	38.6	0.004
Drinking						
Yes	69.8	30.2		55.3	49.1	
No	66.4	33.6	0.03	43.7	50.9	<0.001

The same number of observations were used as identified in Table 1

^a^ *p*-value was obtained from Chi-square test

In the multivariate analysis (Table 4), the association of working hours with SRH among men was only found in the shortest category of working hours (20–35 h) (OR = 2.16, 95 % CI = 1.54–3.02) (Model 1). Similarly, among women, the shortest category of working hours was associated with a higher risk of poor health. These associations disappeared after adjustment, mainly for socioeconomic factors (Model 2). Among women, apart from the category of the shortest working hours, there was an increasing trend of risk of poor health with an increase of working hours. To illustrate, as compared to standard working hours (36–40 h), long working hours of 52–68 h and ≥69 h were both associated with an incremental increase of poor health, shown by ORs of 1.41 (95 % CI = 1.08–1.84) for the former and of 2.11 (95 % CI = 1.42–3.12) for the latter (Model 1). Only the association of ≥69 working hours persisted even after serial adjustments; however, it was substantially attenuated with the addition of socioeconomic factors (e.g. OR = 1.66 (95 % CI = 1.07–2.57) for ≥ 69 working hours) but only slightly attenuated after further adjustment for behavioural factors (e.g. OR = 1.63 (95 % CI = 1.05–2.03) for ≥ 69 working hours). Among other job characteristics, effects of day labour, as compared to a regular job, were significant and persisted across all models. No association was found for work schedule. The associations with job satisfaction were significant for men (OR = 1.34 (95 % CI = 1.11–1.63)) and were borderline significant for women (OR = 1.43 (95 % CI = 0.98–2.08)).

**Table 4:** Multivariate associations of working hours and other work characteristics with poor self-rated health by men and women pooled over seven years (2005–2011) in the Korean Labour and Income Panel Study

	Men			Women		
	Model 1^a^	Model 2^b^	Model 3^c^	Model 1	Model 2	Model 3
	OR (95 % CI)^d^	OR (95 % CI)	OR (95 % CI)	OR (95 % CI)	OR (95 % CI)	OR (95 % CI)
Working hours						
20–35 h	2.16(1.54, 3.02)	1.11(0.80, 1.54)	1.06(0.76, 1.48)	2.15(1.44, 3.22)	0.97(0.65, 1.44)	0.96(0.65, 1.43)
36–40 h	Reference^e^	Reference	Reference	Reference	Reference	Reference
41–52 h	0.91(0.77, 1.07)	0.89(0.75, 1.07)	0.89(0.75, 1.07)	0.92(0.73, 1.16)	1.00(0.76, 1.30)	1.00(0.77, 1.31)
52–68 h	0.98(0.82, 1.18)	0.94(0.77, 1.16)	0.93(0.75, 1.14)	1.41(1.08, 1.84)	1.16(0.85, 1.60)	1.17(0.85, 1.60)
≥69 h	1.18(0.92, 1.50)	1.03(0.79, 1.33)	1.02(0.78, 1.31)	2.11(1.42, 3.12)	1.66(1.07, 2.57)	1.63(1.05, 2.53)
Working days						
≤5		Reference	Reference		Reference	Reference
>5		1.03(0.88, 1.21)	1.03(0.88, 1.20)		0.81(0.64, 1.04)	
Job status						
Regular		Reference	Reference		Reference	Reference
Temporary		0.91(0.65, 1.27)	0.89(0.63, 1.24)		1.37(0.98, 1.93)	1.35(0.96, 1.89)
Day labour		1.62(1.25, 2.11)	1.54(1.18, 2.00)		1.45(0.98, 2.15)	1.42(0.96, 2.10)
Work schedule						
Day work		Reference	Reference		Reference	Reference
Shift/night work		1.04(0.85, 1.27)	1.05(0.86, 1.28)		1.64(1.12, 2.38)	1.64(1.13, 2.39)
Job satisfaction						
Yes			Reference			Reference
No			1.34(1.11, 1.63)			1.43(0.98, 2.08)

^a^Model 1: unadjusted model

^b^Model 2: adjusted for age, income and education as well as covariates listed in Table 3

^c^Model 3: adjusted for smoking, alcohol consumption and job satisfaction additional covariates in Model 2

^d^Association was estimated using generalized estimation equation

^e^Reference indicates a reference category

## Discussion

In general, long working hours were closely associated with SRH, particularly among women. There was suggestive evidence for U-shaped associations; that is, adverse effects at both ends of the categories of working hours: shortest (20–35 h) as well as longest (≥69 h) working hours. The associations were largely attenuated after adjustment for socioeconomic factors but slightly for behavioural factors, suggesting that the associations were partly explained by socioeconomic factors. Our findings on associations for work environment support a role for job satisfaction as a measure of the overall evaluation over one’s working environment, independent of long working hours.

### Methodological considerations

A major strength of this study is the use of a full range of working hours including short working hours, instead of narrowly focusing on overtime. The availability of a relatively wide range of covariates, including working conditions, socioeconomic and behavioural factors, was important to allow examination of certain details. By providing different sets of adjustment covariates, we were partly able to test the separate effects of the factors. Further, compared to cross-sectional data, panel data can provide better estimates by controlling for unobserved heterogeneity and offers gains in establishing causality. Study strengths include the application of DAG, which was used for model selection to identify the minimally sufficient adjustment sets. Although DAG approach is based on the deduction of causal structure among variables under investigation, it is supposed to reduce the degree of confounding and selection bias by better representing the underlying causal relationship [25]. There were some potential limitations to our study. SRH may be too broad to detect specific influences of working hours on health. However, SRH is strongly correlated with objective health status [29] and is used as a relevant single measure, particularly when various health influences are expected to include both physical and mental aspects [30]. Though sample restriction to workers who stayed in employment over all of the 6 study years was motivated by the preference to balanced data over unbalanced data, this might result in ‘the healthy worker survival effect’. In a sensitivity analysis, comparing between those who stayed in the sample and those who did not, a lower proportion of poor self-rated health, longer working hours, more frequent regular job, and higher amount of wage were favourable among the former (data not presented). Selective loss of unhealthy workers consequently may have contributed to underestimates of the adverse impact of long working hours on SRH. Another limitation concerns unmeasured confounding factors such as sleep hours and occupational stress, which could not be assessed due to a lack of available data.

### Interpretation and comparisons with previous studies

Consistent with some previous studies but not all, we found a negative association between long working hours and SRH. The ways through which long working hours affect workers’ health status are yet to be explored. Most obviously, long working hours were closely linked with work demands, thereby leading to an increase of occupational stress and bringing on poor general health. Other explanations include extended exposure to hazardous physical, chemical and psychological factors, and insufficient recovery due to short sleep duration [31]. Whether socioeconomic and behavioural factors modify the association between working hours and health is an important issue, and this study, with serial adjustments across models, showed substantial effects of socioeconomic factors but negligible effects of behavioural factors. This may seem to be inconsistent with the argument that long working hours disrupt health behaviours, which in turn partly mediate the relationship between working hours and health [31, 32]. However, this finding echoes previous studies [33], in which work environment explained a larger portion of socioeconomic differences in SRH than did behavioural factors. This implies that those exposed to both long and short working hours may co-exist with other adverse risks, which are more common to those of lower socioeconomic status. Another explanation may be provided from a data construction point of view; among workers who maintained paid employment for long years as seen in our sample, the adverse effects of long working hours through the behavioural path may be minimized for adaptation. This suggests that enabling people to stay in employment may help to promote healthy behaviours, which may be particularly prominent among women.

The worsening of SRH with increasing working hours was found only among women. This finding agrees with previous studies [2, 3, 34], including a study from the same population [35], which was limited to a cross-sectional analysis from one wave of the KLIPS. Two hypotheses can be addressed for explanation. First, female workers may be under the ‘double burden’: family and work responsibilities [36]. Though the hypothesis was not tested in the current study, in a study from Korea, female workers took most of the household tasks after work time [37]; women thereby became more susceptible to experiencing health problems. Second, this observation may be rooted in the unequal power relation between men and women, which consequently is reflected in the labour market position. Women often worked in more precarious and subordinate positions than men, as broadly shown in the current study (Tables 1 and 2) and other studies [38]. This subsequently leads to more exposure to hazardous and unfavourable working conditions towards women’s health [39]. However, the gender difference may also be because of the health measure used in this study; that is SRH. Women tend to report poorer SRH than men across countries and age groups [40], though the evidence on whether women’s assessment of their own health relative to their actual health status is more negative than men is unclear [41, 42]. Putting things together, long working hours may have a greater negative impact on women than men under the same conditions.

Interestingly, our study suggests that both the shortest as well as the longest working hours are associated with poorer SRH, resulting in a U-shaped association. This pattern, though not conclusive, was similarly observed in a meta-analysis for coronary heart disease [43], while other studies reported a gradual increase of poor health in response to an increase of working hours [44, 45], where working hours shorter than a standard category was mostly neglected. Reverse causation may be plausible. Despite the longitudinal nature of this study, poor health might affect the ability to work [23], as participants were not free from poor health at baseline. Thus, some of those who work short hours do physically light work because of health reasons, though such findings remain to be explored. This, together with another finding that the association was markedly attenuated by further adjustment for socioeconomic factors, implies that there has been bi-directional accumulation between poor health and lower socioeconomic status; those who work short hours do so as more of their socio-economical vulnerability than as of voluntary choice [46]. More attention in future studies needs to be paid to cover a wider range of working hours as the atypical nature of working hours is increasing at both ends of the range. A potential independent association of job satisfaction is also important. Job satisfaction represents the subjective combination of job security, control over workload, and levels of enjoyment [47]. Since the concept relates to a general condition rather than domain specific, job satisfaction is expected to be of potential importance for workplace policies aimed at improving employee’s global health status.

## Conclusion

Long working hours were associated with a higher level of poor health. The impact was larger among female workers, who frequently continue to manage the demand of double role in family and work. The health risk of long working hours was partly explained by socioeconomic condition. In sum, this study addresses that the reduction in working hours could be a better measure for labour protection, when coupled with improvements in other adversities tied to gender and socioeconomic status (i.e., traditional gender role and precarious job characteristics).

## Additional file

Additional file 1:
**Directed acyclic graph illustrating the hypothesized pathway of working hours to self-rated health and associated covariates.** (PDF 81 kb)

## Abbreviations

CI: Confidence interval
DAG: Directed Acyclic Graph
KLIPS: The Korean Labour and Income Panel Study
OR: Odds ratio
SRH: Self-rated Health
