# Supplementary figures and images for: Working hours and self-rated health over 7 years: gender differences in a Korean longitudinal study
Source: BMC Public Health. 2015 Dec 23;15:1287. doi: 10.1186/s12889-015-2641-1 (PMC4690406; doi:10.1186/s12889-015-2641-1)

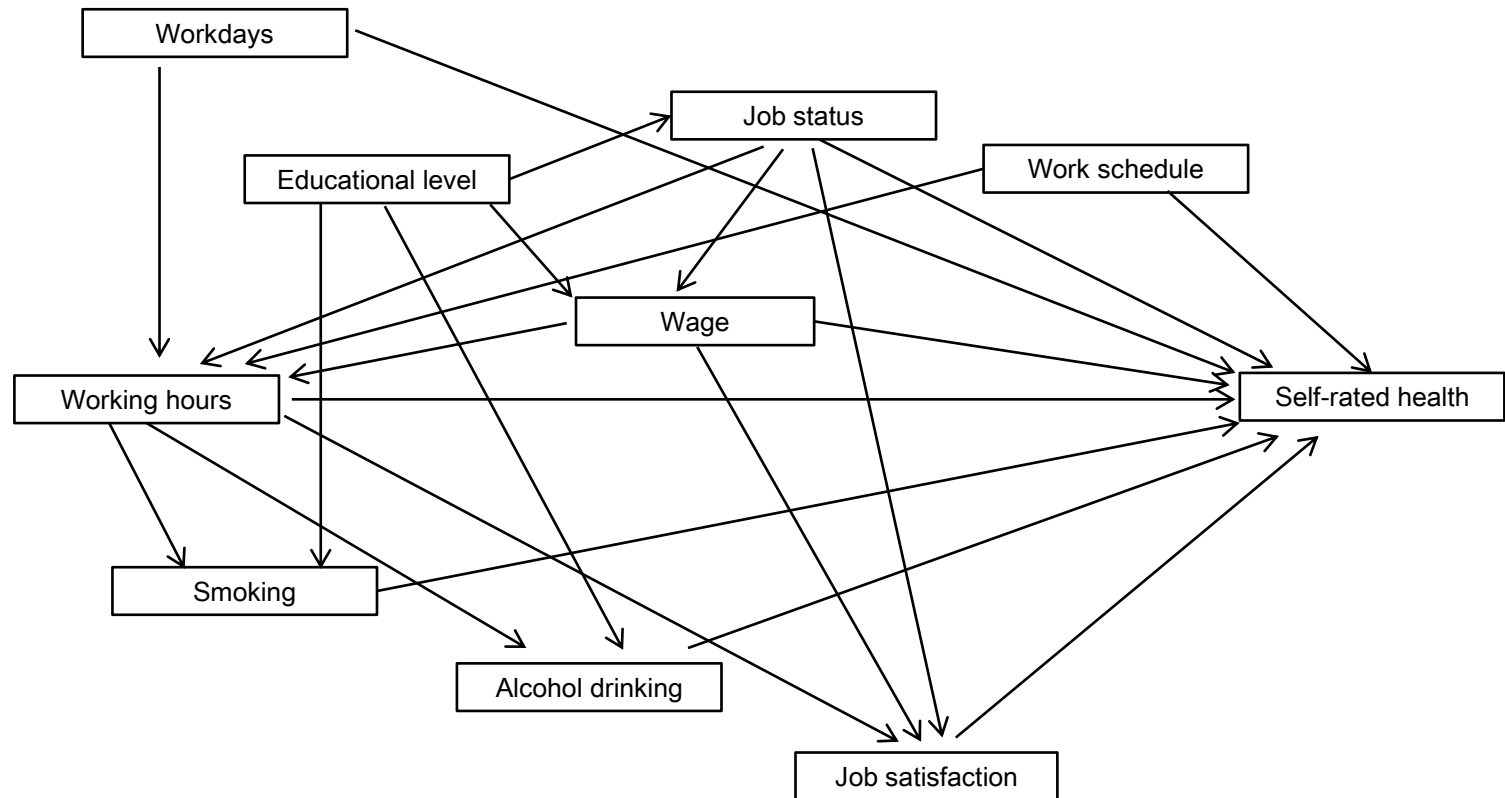

Supplement: Additional file 1: — Directed acyclic graph illustrating the hypothesized pathway of working hours to self-rated health and associated covariates. (PDF 81 kb) [file 12889_2015_2641_MOESM1_ESM.pdf]
